# Integration of safety and sustainability criteria into early design stages of battery cell manufacturing machinery

**DOI:** 10.1038/s41598-025-30510-7

**Published:** 2025-12-19

**Authors:** Cristina Cerrillo, Gemma Mendoza, Guillermo Ormazabal, Vladimir Popok, Heiko Oetting, Benedikt Konersmann, Alessandro Tedeschi Gallo, Maeva Lavigne Philippot

**Affiliations:** 1 Tekniker, Basque Research and Technology Alliance (BRTA), C/ Iñaki Goenaga, 5, 20600 Eibar, Spain; 2 FOM Technologies, Kastrup, Denmark; 3 NETZSCH Feinmahltechnik, Selb, Germany; 4 RWTH AACHEN UNIVERSITY, Aging and Lifetime Prediction of Batteries, Institute for Power Electronics and Electrical Drives (ISEA), Campus-Boulevard 89, 52074 Aachen, Germany; 5 Helmholtz-Institute Münster (HI MS), Institute of Energy Materials and Devices (IMD-4), Forschungszentrum Jülich GmbH, Campus-Boulevard 89, 52074 Aachen, Germany; 6 Deep Blue, Rome, Italy; 7 Electromobility Research Center (MOBI), Department of Electric Engineering and Energy Technology (ETEC), Vrije Universiteit Brussel, Pleinlaan 2, 1050 Brussels, Belgium

**Keywords:** Safe and sustainable by design (SSbD), Battery cell production, Battery manufacturing machinery, Slurry mixer, Slot-die coating, Roll to roll (R2R), Calendaring unit

## Abstract

There is currently a lack of guidance on implementing safety and sustainability at early design stages of battery cell manufacturing machinery. In this work, key European regulatory requirements for battery manufacturing and relevant Safe and Sustainable by Design (SSbD) approaches to lithium-ion battery (LIB) cell production and machinery were explored. Considering peer-reviewed publications and European projects, it was found that the existing literature on the implementation of the SSbD criteria in battery production is still scarce, particularly concerning machinery design. In our paper, data on the current design principles were collected from two battery machinery manufacturers covering, for the first time, the main processes for LIB electrode production (mixing, coating, drying and calendaring), and key areas to formulate SSbD practice guidelines were identified. The origin, hazards and criticality of materials, and the possibility of being renewable or recycled are crucial in terms of raw materials selection. For the manufacturing stage it is recommended minimizing the number of production processes, energy consumption and waste generation, using renewable energy sources, and implementing easy assembly processes and automation. Considering a modular design and the traceability of the machinery components will facilitate maintenance during the use stage, adaptation to new user requirements and disassembly and recycling at the End-of-life.

**Supplementary Information:**

The online version contains supplementary material available at 10.1038/s41598-025-30510-7.

## Introduction

The global battery demand is pushing for safe and sustainable manufacturing value chains. The next generation of battery manufacturing processes should incorporate scalable, digital and energy-efficient techniques. Developments should cover the entire value chain (slurry mixing, coating and drying, calendaring and battery cell production), using machinery that minimizes the energy for cell production, enhances plant efficiency rates, increases flexibility regarding changes on battery formulations, and integrates intelligent control processes to reduce scrap. The European Union has progressively established a regulatory framework for safety and sustainability requirements on batteries. The Ecodesign Directive (2009/125/EC) set a baseline for sustainable product design across industries and energy-related products to make them more energy-efficient^1^. Building on this foundation, the Ecodesign for Sustainable Products Regulation (ESPR) was proposed as a more comprehensive update^2^. It applies to a wider range of products, including batteries, and sets new requirements for durability, repairability, recyclability, and efficient resource use. The ESPR introduced the Digital Product Passport (DPP), a digital tool designed to provide lifecycle and sustainability information across industries, making product traceability and resource management more transparent. Furthermore, the Battery Regulation (2023/1542) addresses the environmental and social impacts of batteries throughout their lifecycle^3^. It sets requirements for safe and sustainable production, use, and disposal of batteries, and establishes batteries as the first product group for which the use of a DPP will be a legal requirement as of 2027. This Regulation also establishes information that will be mandatory on the percentage share of cobalt, lithium or nickel present in active materials that have been recovered from battery manufacturing waste or post-consumer waste. In addition, the Green Deal Industrial Plan introduced by the EC in February 2023 encompassed initiatives such as the Net-Zero Industry Act (NZIA) and Critical Raw Materials Act (CRMA), which are also expected to have a direct impact on battery sustainability^4^.

Particularly, the EC Joint Research Center (JRC) has developed a framework for the definition of SSbD criteria and evaluation procedures^5^. It aims to support the design and development of safe and sustainable chemicals and materials within the research and innovation (R&I) stage. The definition of SSbD in this framework considers:

- A pre-market approach to chemicals and materials design that focuses on providing a function (or service).
- Avoiding volumes and chemical and material properties that may be harmful to human health or the environment.
- Ensure overall sustainability by minimizing their environmental footprint on climate change, resource use, and protecting ecosystems and biodiversity, from a lifecycle perspective.

The existing literature on the implementation of the SSbD approach in battery production is still scarce, particularly regarding the machinery design stage. In terms of peer-reviewed papers, recent publications on sustainable practices for lithium-ion battery (LIB) manufacturing deal mostly with performance improvement using digitalization tools, such as machine learning^6–8^. Only the investigation by Soeteman-Hernández et al.^9^ offers guidance for a broad SSbD implementation to the next generation of battery technologies. They recommend a Life Cycle Thinking (LCT) approach, the integration of safety and social, economic and environmental assessments for supply chains, and addressing ‘‘by-design’’ in addition to just a safety and sustainability assessment. These authors support especially the new understanding of sustainability, safety and the applicability of SSbD provided by the European Chemical Strategy for Sustainability (EC-CSS)^10^. The very few studies found on the application of the SSbD approach to the design of the machinery address only one or two of the sustainability pillars. For instance, a quality assurance concept based on statistical process controls and a digital twin of the machine was proposed to reduce the iterations during the design stage and hence the economic impact of the machine development^11^. Other authors considered the machinery design in the development of an electrode production process and achieved increased energy efficiency of the drying, CO_2_-footprint reduction, and the absence of sedimentation effects^12^. Machine availability and site locations have also been regarded to assess energy consumption in the cell manufacturing process, and reductions of 13–30% were observed depending on the machine availability scenario^13^.

The scope of the state-of-the-art in this study was extended to other publications in the context of the expertise of the battery research community in Europe. The last R&I Roadmap presented by Batteries Europe^14^ aimed at an SSbD framework for batteries development going beyond regulatory compliance and supporting the development of an integrated value chain. Specific activities needed in the short (2027) and mid-term (2030) concerning the SSbD considerations in the design stage of the machinery were proposed as well:

- Improve machines and implement systems to reuse energy (e.g., recirculation of electrical energy to charge/discharge cells, reuse of thermal energy in the plant).
- Development of processes that can use state-of-the-art machines to enable a smooth transition to new technologies. Li-ion and Na-ion have similar production processes, redox flow batteries (RFB) and solid state may require specific developments.
- As there will be a range of new materials introduced into existing processes for electrodes and cells, a comprehensive understanding of the relationship between materials, cell designs, and production processes is needed to facilitate development of interchangeable and modular equipment.
- Standardized data interfaces to facilitate the integration of new machines into existing lines and the monitoring and control of production processes.
- Implementation of digital twins to rapidly scale up machines and production lines.

More recently, the Strategic Research and Innovation Agenda published by BEPA and Batteries Europe^15^ included these research actions in terms of the design stage of the machinery:

- Multi-purpose production line development, encompassing machinery compatible with diverse chemistries, cell format/design, and process technologies, and pilot-level validation and simulation to assess applicability to mass production scale.
- Enhance the capacity of LIB giga-factories to accommodate new chemistries and advanced production concepts and machinery, and fortify production lines to withstand disruptive scenarios, ranging from energy source variability to supply chain disruptions. Conduct sensitivity studies on current equipment to assess their response to different scenarios, followed by designing potential tools or upgrades for improved resiliency and adaptability.

Consequently, the strategic agendas and roadmaps available propose general considerations on the R&I activities needed, rather than specific guidance for the machinery design.

In addition, the SSbD approach is being tackled in European projects on battery production currently underway. Sustainability aspects of batteries have been studied by some projects running prior to the recommendation by the European Commission for a SSbD framework^16^. Other have started after the EC’s recommendation addressing the SSbD approach to a certain extent, and there are currently some ongoing projects aiming at its full application covering machinery as well. This evolution is presented in Table 1 based on the public information available, including the projects found and their main topics.

**Table 1:** Evolution on the application of the SSbD approach in the European projects on LIB cell production and machinery.

Starting year and SSbD criteria application level of the projects	Acronyms and reference	Main topic
Projects started between 2012 and 2022 (prior to the EC recommendation), considering specific sustainability aspects of batteries	ELCAR^17^	LCA guidelines for BEV
	STORY^18^	LCA and (non-comprehensive) S-LCA of ESS
	BAHLIT^19^	Organic redox flow batteries
	HELIOS^20^	Performance, circularity and sustainability of urban mobility models based on BEV
	BATWOMAN^21^	Sustainable and cost-efficient solutions for LIB cell production
	GIGAGREEN^22^	Improving the environmental, economic and social performance of 3b generation Li-ion cells
	GREENSPEED^23^	Development of a new cell based on Ni-rich NMC with increased energy density
	NOVOC^24^	Cell-manufacturing technologies with reduced costs, avoiding the use of toxic organic solvents
	BATRAW^25^	Safer and more efficient battery recycling processes
Projects started between 2023 and 2024 (after the EC recommendation), considering the SSbD approach to a certain extent	RENOVATE^26^	Closed-loop processes for recycling EoL batteries
	REUSE^27^	Sustainable recycling processes for low-value LFP battery waste
	REVITALISE^28^	Low-cost and low environmental impact recycling of NMC, LFP and Na-Ion batteries
	LI4LIFE^29^	Boost the EU’s domestic lithium supply
	SAFELOOP^30^	Safety, sustainability, and performance of EU’s Gigafactory scale LIB cells
	RECIRCULATE^31^	Blockchain-based platform to improve the reuse and recycling of battery value chain
	TRANSENSUS LCA^32^	Harmonization of LCA, S-LCA and LCC approach for a zero-emission road transport system in Europe
Projects currently underway applying the SSbD approach	STREAMS^33^	Technologies for sustainable production of precursors and active materials
	SUESS^34^	Application of SSbD approach to selected ESS (flow batteries and LIB)
	INERRANT^35^	SSbD materials and processes for LIB in electromobility applications
	SOLVE^36^	SSbD prototypes for mass production of Solid-state batteries 4b generation
	IRISS^37^	SSbD roadmaps development for seven value chains in collaboration with industry, including batteries
	BATMACHINE^38^	Advanced battery cell manufacturing machinery for sustainable processes
	GIGABAT^39^	Sustainable 3b generation LIB and energy-efficient machinery for gigafactories
	SIERRA^40^	Safe, sustainable, lightweight and high-performance Ni-rich NMC battery pack

3b generation = Optimised Li-ion; 4b generation = Solid state Li metal.

The projects BATMACHINE and GIGABAT were funded under Horizon Europe call for proposal HORIZON-CL5-2022-D2-01-04 (Towards creating an integrated manufacturing value chain in Europe: from machinery development to plant and site integrated design)^41^. They address specifically the design stage of the machinery, and valuable insights on SSbD strategies should be derived from these projects in the near future. Moreover, the project SIERRA was funded under the call HORIZON-CL5-2024-D2-02-03 (Size & weight reduction of cell and packaging of batteries system, integrating lightweight and functional materials, innovative thermal management and safe and sustainable by design approach)^42^. This project started in June 2025 and aims clearly to safe and sustainable high-energy NMC cells. As can be ascertained, the SSbD approach is being considered progressively in European projects. However, public reports addressing in an exhaustive manner the design of safe and sustainable batteries and manufacturing machinery have not been delivered so far.

This research primarily focuses on evaluating how SSbD can be integrated into the machinery design stage. Safety and sustainability measures are analyzed early in the equipment development process to minimize the use of materials that might have an impact on human health and the environment. The aim is to provide adapted guidelines to the battery manufacturing industries for further development.

## Materials and methods

### State of art on SSbD challenges and actions for LIB cell production and machinery

In view of the scarcity of studies focused on machinery design, the state of art covered both design of machinery and the overall cell manufacturing process. Especially for peer-reviewed papers, very few results were obtained in the searches conducted, so the scope was extended to other publications from the battery research community. The search tools and keywords used are described below.

- Several searches of peer-reviewed papers were conducted on the Web of Science^43^. See Supplementary Information for details on search parameters.
- For European projects, a search in CORDIS^44^ was performed, and other additional sources were considered based on previous experiences and projects in which the authors are involved. The search terms entered in CORDIS were “*safe and sustainable by design*” and “batter*”, and they were filtered by the “*Projects*” Collection.

### EC JRC SSbD framework and SSbD design principles

**Fig. 1:**
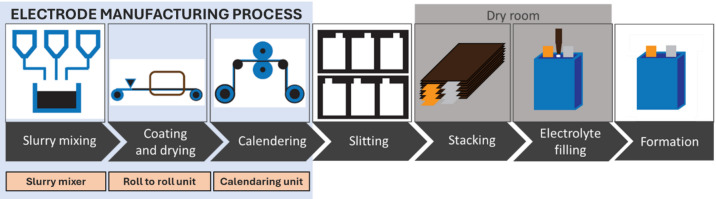
Overview of the main processes and machinery for electrode production within the LIB cell manufacturing steps (adapted from^45^).

In this study, data was collected from two battery machinery manufacturers (FOM TECHNOLOGIES and NETZSCH Feinmahltechnik GmbH) on their current design principles. The focus was placed on different machinery representative of the main steps in the electrode manufacturing process: Slurry mixer with mill, Roll to roll (R2R) for electrode coating and drying, and Calendaring unit (Fig. 1). Although subsequent steps in the battery cell production can also be relevant in terms of safety and sustainability, the machinery under development in the BATMACHINE project^38^ was selected. This machinery was prioritized to reduce energy consumption, scrap-rates and production costs with more efficiency (less downtime, higher throughput and a modular setup), to improve product quality, and to follow universality concepts increasing flexibility and compatibility with other electrode materials.

**Fig. 2:**
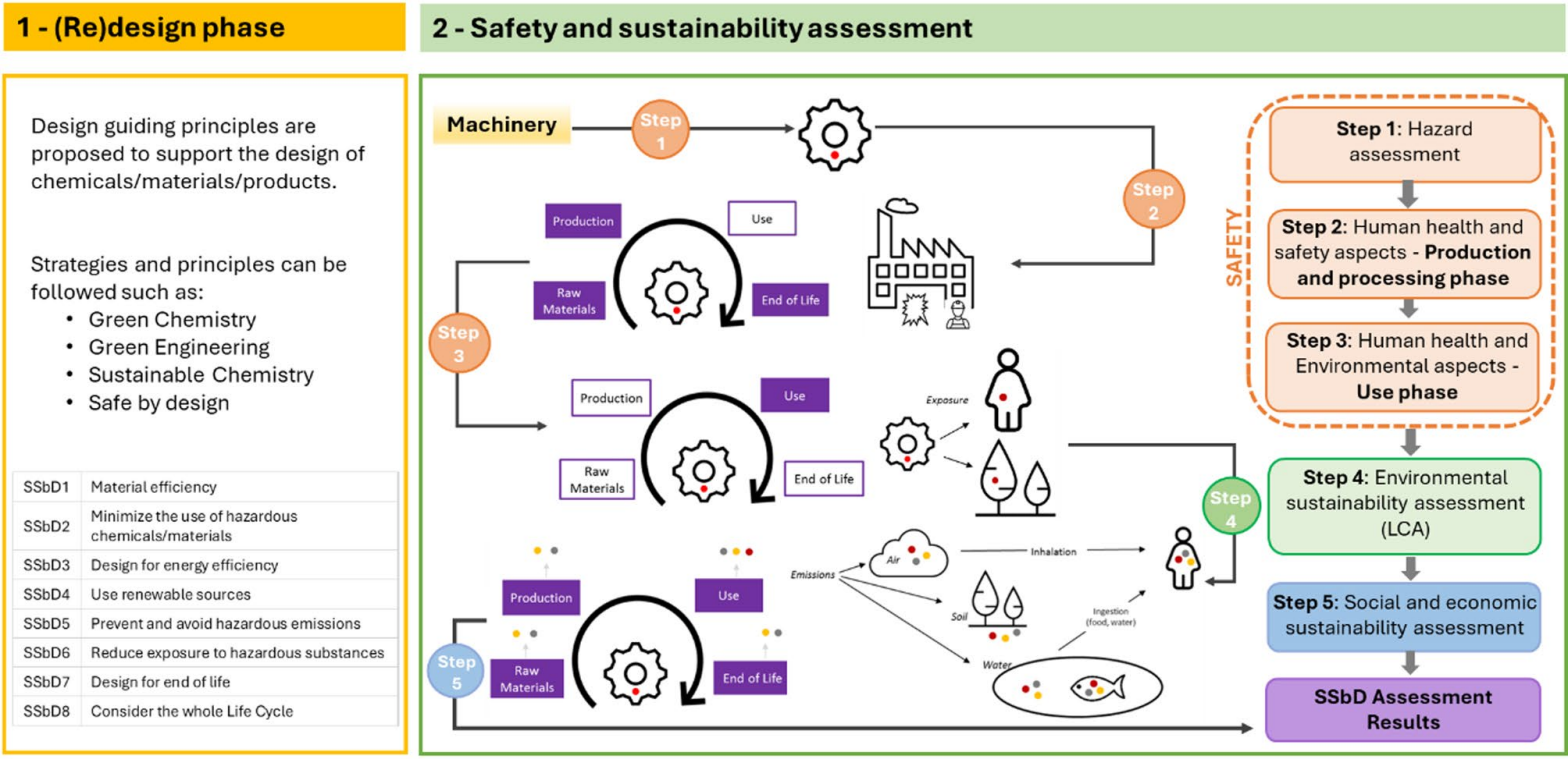
Stepwise approach for the SSbD framework safety and sustainability assessment, adapted from Figs. 9, 10 and 11 in the original source^5^. The red dot refers to the chemical/material under evaluation while the orange/grey dots refer to all the other substances emitted along its life cycle (e.g. other toxic chemicals emitted in extraction of raw material or due to the energy use in manufacturing).

Then, key areas were identified to formulate SSbD practice guidelines for the machinery, considering the SSbD design principles proposed by the EC JRC framework to be adopted in the (re)design phase. The JRC SSbD framework recommends a two-phase approach^5^, represented in Fig. 2 below:

- First, a (re)design phase in which several guiding principles are proposed to support the design of chemicals and materials,
- and then a stepwise hierarchical approach is proposed to address the comprehensive safety and sustainability assessment of the new designs by carrying out different analyses on chemical safety, direct toxicological or ecotoxicological impact, aspects of environmental sustainability, as well as social and economic conditions.

A specialized questionnaire was developed in the form of a checklist by adapting the eight SSbD guiding principles in Fig. 2 to the needs of the battery machinery (Table 2). The checklist was organized in four different sections addressing the entire life cycle of the machinery, and was derived from the SSbD principles, their associated definition, and the examples of actions for the design phase (provided in Supplementary Table S1). Annex 2 in the framework including the complete list of underpinning principles and approaches was also used to develop this questionnaire. Since the guidance on the design principles provided in the framework is illustrative and not exhaustive, it was necessary to adapt and expand the list of principles according to the specific design purposes of the machinery. For instance, the reduction in the number of different material types (position 1.7 in Table 2) was proposed based on the minimization of the number of chemicals in the production process (under SSbD principle number 1 in Supplementary Table S1) and on the minimization of material diversity (Green Engineering principle number 9 in Annex 2 of the framework). The avoidance of permanent joints in the design (position 4.4) pursued the selection of materials that are easy to separate and sort, corresponding to SSbD principle number 7 in Supplementary Table S1 and the Green Engineering principle number 3 in Annex 2. Prior to the evaluation, the manufacturers approved the checklist items to ensure their applicability to assess the machinery design and how SSbD criteria could be integrated.

**Table 2:** Checklist with SSbD guiding principles proposed for the battery machinery design stage.

Section 1	Design stage-Materials efficiency—Ecodesign strategies (SSbD1; SSbD2; SSbD4, SSbD5)
1.1	Use of renewable/biobased materials in battery machinery
1.2	Use of recycled materials
1.3	Consideration of the future recyclability of materials during the selection
1.4	Use of low energy content materials
1.5	Consider environmental criteria when selecting suppliers
1.6	Prioritize the use of local raw materials
1.7	Reduce the number of different material types
1.8	Avoid overdimensioning by implementing mechanical analysis
1.9	Reduction of materials usage (Reduction in weight, volume)
1.10	Avoid the use of hazardous materials
1.11	Avoid the use of Critical Raw Materials (CRM); see Fifth list 2023 of CRM for the EU in Supplementary Table S2, and full report in^46^
Section 2	Manufacturing stage—Design for energy efficiency (SSbD3), prevent and avoid hazardous emissions (SSbD5) and reduce exposure to hazardous substances (SSbD6)
2.1	Reduce the number of production processes
2.2	Use of renewable energy
2.3	Optimize the energy consumption
2.4	Consider easy assembly to automate assembly processes (and subsequent dis-assembly)
2.5	Analyse new fabrication processes, optimization the process
2.6	Inspect the acceptance of the finished product at the factory
2.7	Reduce the number of auxiliaries and operational materials (e.g., water, oil, solvents)
2.8	Minimize waste production in manufacturing
2.9	Implement proper waste management in manufacturing
2.10	Use sustainable packaging trying to minimize the quantity
2.11	Use renewable transportation alternatives and optimize the logistic
Section 3	Use stage—Design for energy efficiency (SSbD3) and durability, prevent and avoid hazardous emissions (SSbD5) and reduce exposure to hazardous substances (SSbD6)
3.1	Consider the safety of the technicians
3.2	Avoid the generation of hazardous emissions during use
3.3	Minimize the number of connections in the equipment
3.4	Reduce energy consumption in comparison to similar products
3.5	Use clean energy
3.6	Establish a modular and scalable design so that it can adapt to new user requirements (e.g., compatibility with diverse cell chemistries)
3.7	Use modular assemblies that allow for the replacement of critical components. Design considering easy access to parts likely to need maintenance
3.8	Sensorisation of the product to more effectively identify the source of faults
3.9	Use digital twins to predict and correct the proper functioning of the product
3.10	Reduce the number of consumables
3.11	Choose consumables with low environmental impact
3.12	Optimization of the reliability and durability of the product
3.13	Consider whether it is necessary to sell the product or charge for its use (servitisation of the product)
Section 4	End-of-life—Consider the whole life cycle including the EoL (SSbD7, SSbD8)
4.1	Build the equipment in a modular way to facilitate maintenance and recycling at the EoL
4.2	Design the system considering that the tools required for disassembly are available and simple
4.3	Reduce the number of tools required for disassembly
4.4	Avoid permanent joints in the design
4.5	Standardize the different machine components so they can be reusable
4.6	Use surface treatments that are easy to remove
4.7	Recovery of recyclable materials (see UNE-EN ISO 11469:2001)
4.8	Minimize the landfill and incineration waste generation

The evaluation of the current principles applied was conducted by the machinery manufacturers, using a score system based on the degree of consideration of each principle, by assigning values from 0 to 3 (where 0 = not any consideration, 1 = little, 2 = good, 3 = excellent). To minimize subjectivity by the manufacturers and ensure consistency in the evaluation, a detailed definition of each value was provided in specific meetings with them as follows:

- Score “0”: No action is taken yet.
- Score “1”: First actions to address this principle have already been taken.
- Score “2”: There is evidence (e.g. sustainability reports) that the company has started to implement this principle with a clearly defined timeline.
- Score “3”: The progress in the implementation of this principle is beyond compliance with local and/or international standards (if any). The manufacturer has committed itself to be best in class regarding the performance on this principle, and there is audited evidence of its implementation.

The description of this scoring was based on the reference scale approach recommended by the JRC SSbD framework to assess the performance of system/organisations’ activity^5,47^, and on the guidance provided by UNEP^48^ and the Social Value Initiative^49^ to develop reference scales.

Benchmark scores were proposed as well for each principle to enhance the understanding of the current progress in their application to machinery. These baseline scores were set as the lowest values assigned to each principle by the manufacturers.

Finally, the results obtained in this evaluation were analysed to find hotspots and to formulate practice guidelines based on the JRC SSbD framework design principles.

## Results and discussion

The scoring assigned to each design principle by the machinery manufacturers is provided below in this section, and the comparative analysis with benchmark values is included at the end to better ascertain the degree in the adoption of each principle.

### Slurry mixer with mill case study

Section 1 dealing with the design stage, materials efficiency and ecodesign strategies adopted, obtained the lowest evaluation for the mixer and mill design principles (Table 3). This result is due to the fact that renewable, recyclable, recycled, and biobased materials are not used in the design stage, together with the use of hazardous materials and CRM. One case of not using recycled materials is, for example, the requirement of purchasing certificated stainless steel, which does not contain a recycled portion. It would be recommended to know from the supplier which are these specific requirements and if the use of recycled material is a feasible option, as well as considering new suppliers which can provide products with recycled material in their catalog.

**Table 3:**
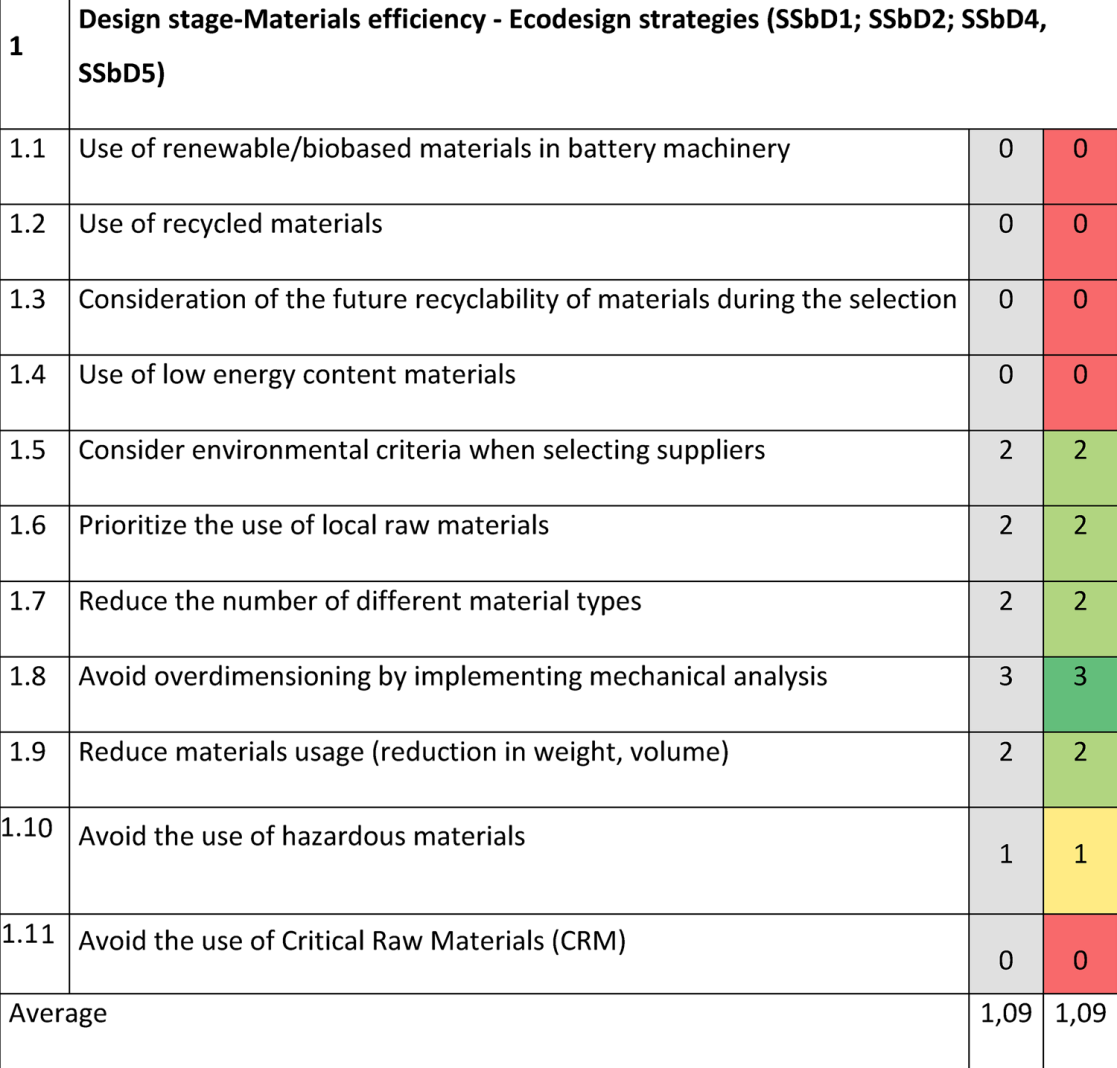
SSbD guiding principles evaluation for the Slurry mixer with mill in Section 1, performed by NETZSCH Feinmahltechnik GmbH.

The scoring is assigned in the colored cells based on the degree of consideration, where 0 = not any consideration, 1 = little, 2 = good, 3 = excellent. Benchmark values are provided in the column shaded in grey.

Regarding the use of CRM, it would be advised to identify which of them is present in the machinery, specifying which component or process they are involved in and their role. Once the CRM and their functions have been identified, some actions would be necessary to avoid their use or at least try to reduce them if a complete avoidance is not possible, always taking into account not affecting the functionality and correct performance. The use of some CRM is indeed essential in the manufacturing of machinery and equipment, as established in the last study for the EU^46^.

Additionally, the hazard assessment proposed by the SSbD framework is recommended concerning the use of hazardous materials. It is addressed in Step 1 of the safety and sustainability assessment, which corresponds to the second phase of the SSbD framework (Fig. 2). It aims to identify the most appropriate criteria that can be applied during the (re)design of chemicals and materials, in order to align with the overall objectives of the EC-CSS, including the definition of the aspects, indicators, criteria and an evaluation system.

This methodology defines three main categories of aspects: intrinsic hazard properties relevant to human health (human health hazards), intrinsic hazard properties relevant to the environment (environmental hazards) and physical properties (physical hazards). As information becomes available along the life cycle of a chemical/material, the aspects that need to be evaluated to fulfil the criteria of Step 1 are also detailed in the JRC SSbD framework (Supplementary Table S3). The grouping of the hazard properties is aligned with and follows relevant EC initiatives, such as the EC-CSS and Classification, labelling and packaging (CLP) regulation, the proposal for a regulation regarding sustainable products, or the EU Sustainable Finance, all of them referenced in the JRC SSbD framework^5^. Based on those hazard properties, three criteria (H1, H2 and H3) are defined including descriptions, observations and some recommendations about how to proceed respecting these criteria (Supplementary Table S4). Although the applicability of Step 1 on the battery manufacturing machinery might seem specific for chemicals or materials, according to the SSbD framework definitions the safety concept is transversal to all sustainability dimensions, and when applied in the context of chemicals/materials, sustainability covers also products and services. In addition, in the context of SSbD criteria definition for chemicals and materials, the term ‘*by-design*’ can be interpreted at 3 levels: molecular design, process design, and product design (the design of the product in which the chemical/material is used). It is also intended to be useful for product designers, when they need e.g. to select different chemicals and materials to meet the functional demands of the product under development.

**Fig. 3:**
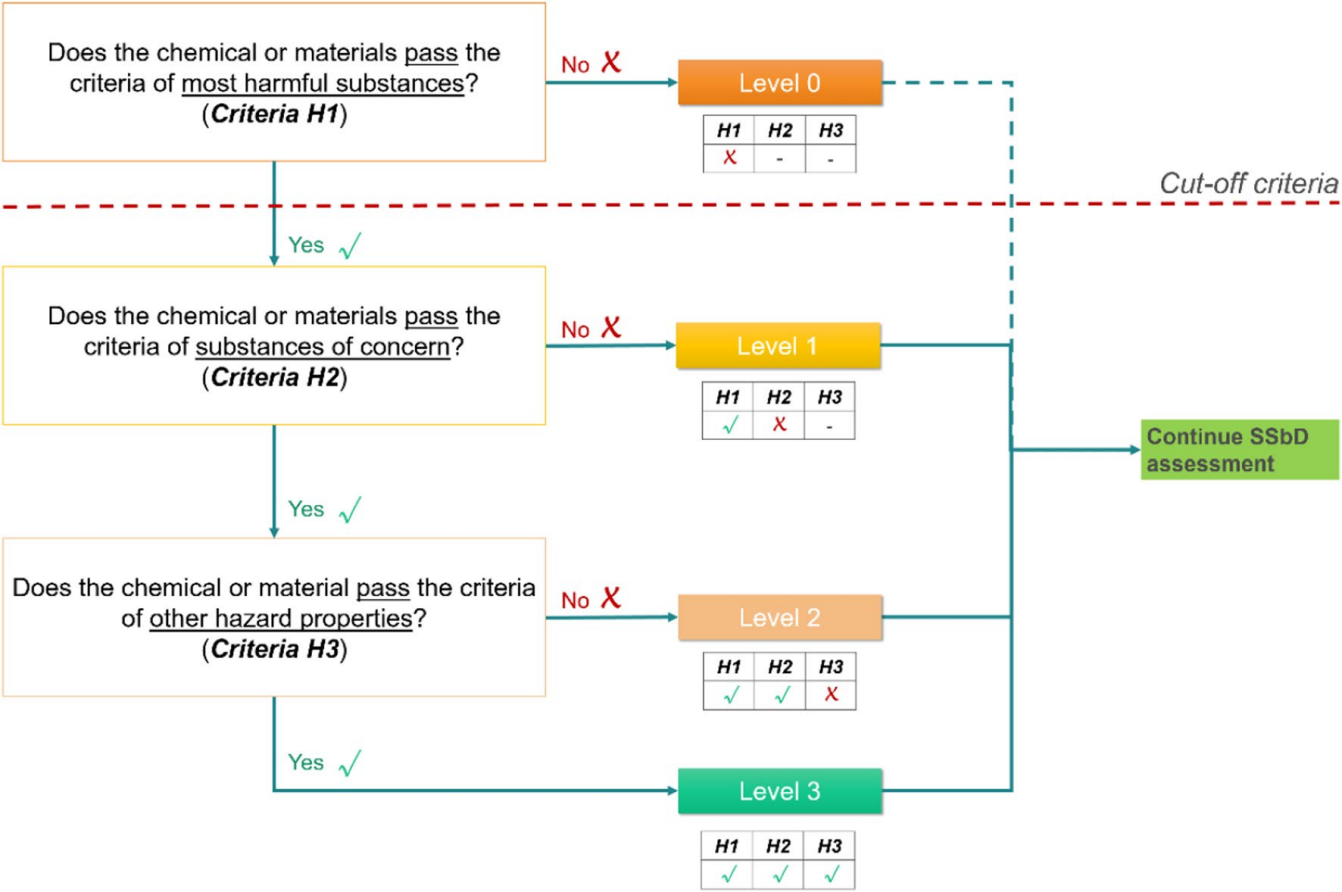
Workflow relevant to Step 1 of the JRC SSbD framework^5^.

According to this evaluation system, the chemicals or materials that pass a certain criterion of Step 1 will get a ‘level’ that reflects the result of the hazard assessment related to aspects included in that specific criterion. For SSbD Step 1, four levels were envisioned (from ‘Level 0’ to ‘Level 3’) to allow the assessor to rank a specific chemical based on these levels and further to integrate the results of the hazard-based evaluation to the overall SSbD assessment (Fig. 3). For Level 3 chemicals or materials considered of no concern regarding intrinsic hazard properties, it should be recognized that they could still pose harm in certain applications from a risk perspective that goes beyond generic hazard criteria and includes consideration of application-specific exposure settings.

Concerning Section 2 focusing on the manufacturing stage, design for energy efficiency, prevention and avoidance of hazardous emissions and reduction of exposure to hazardous substances, a better evaluation was shown for the mixer and mill design principles, with an average score of 2,00 (Table 4). Minimum scores corresponded to the reduction of the number of production processes (position 2.1) and the optimization of energy consumption (position 2.3), which are especially related to the SSbD3 design principle. Considering the examples of actions in Supplementary Table S1, some improvements can be recommended in specific areas, such as:

- optimizing energy efficiency of manufacturing processes,
- maximising energy re-use (e.g., heat networks integration and cogeneration),
- reducing production steps (e.g., applying lean thinking),
- reducing inefficiencies and exploit available residual energy in processes.

**Table 4:**
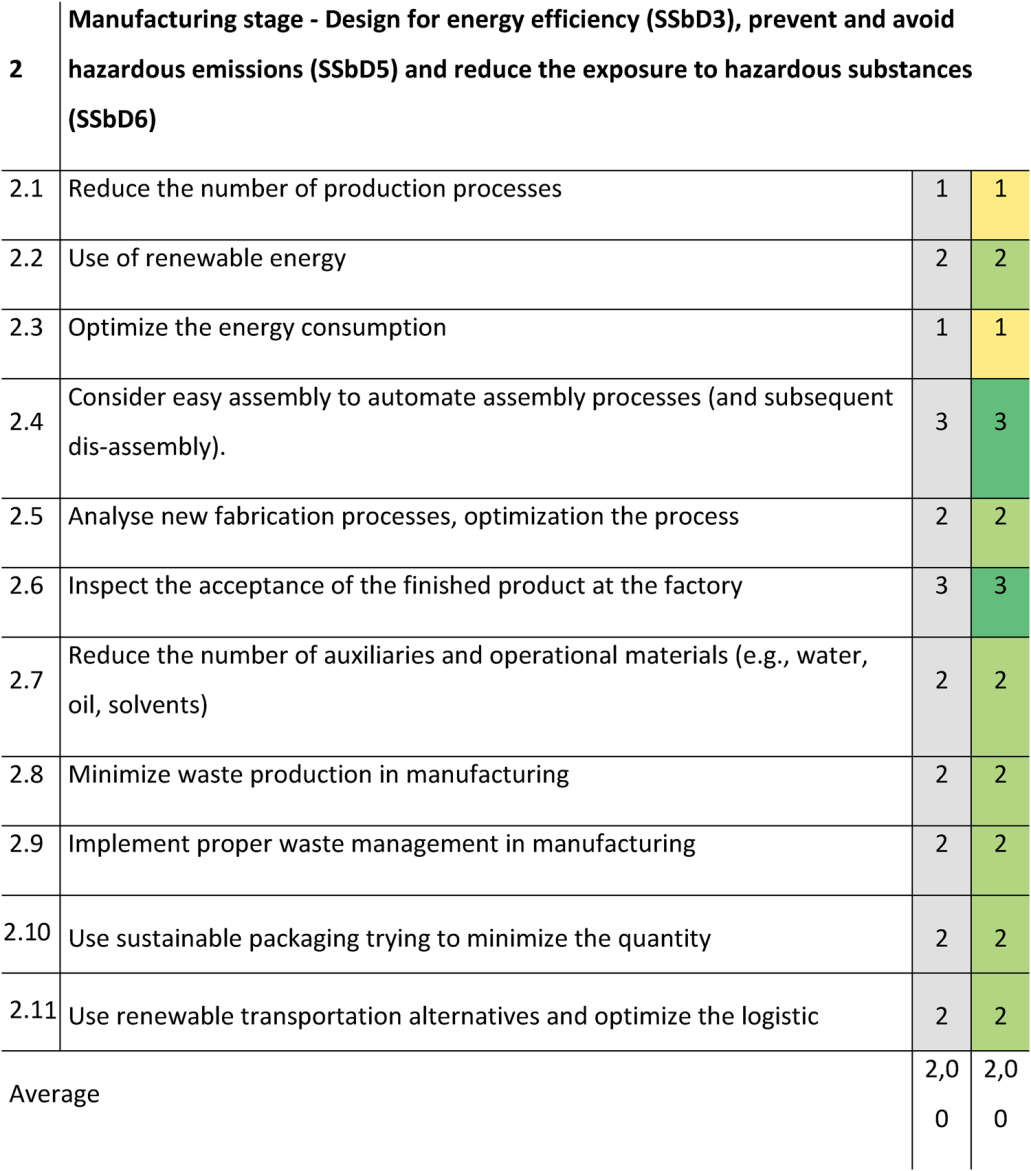
SSbD guiding principles evaluation for the Slurry mixer with mill in Section 2, performed by NETZSCH Feinmahltechnik GmbH.

The scoring is assigned in the colored cells based on the degree of consideration, where 0 = not any consideration, 1 = little, 2 = good, 3 = excellent. Benchmark values are provided in the column shaded in grey.

Section 3 of the evaluation, related to the use stage and linked to the same principles as Section 2 (SSbD3, SSbD5 and SSbD6), was the best evaluated with an average score of 2,54 (Table 5). Although there is a poor evaluation about the use of digital twins to predict and correct the proper functioning of the product (position 3.9), it seems to be a very optimistic goal, which would be preferable to consider in the long term. It might be easier to act on reducing the number of consumables (position 3.10) or choosing consumables with low environmental impact (position 3.11).

**Table 5:**
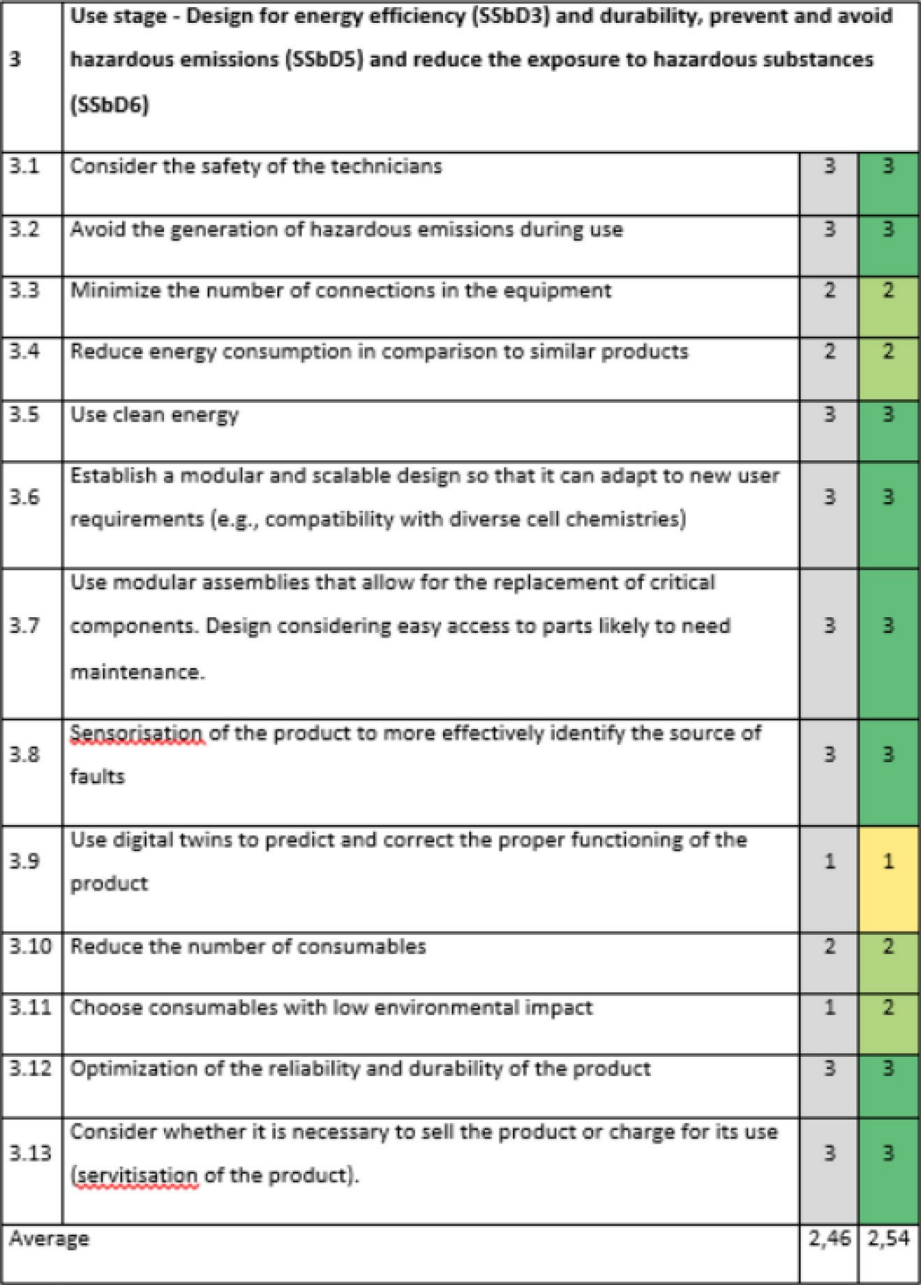
SSbD guiding principles evaluation for the Slurry mixer with mill in Section 3, performed by NETZSCH Feinmahltechnik GmbH.

The scoring is assigned in the colored cells based on the degree of consideration, where 0 = not any consideration, 1 = little, 2 = good, 3 = excellent. Benchmark values are provided in the column shaded in grey.

In Section 4 addressing the EoL stage (Table 6), an average score of 2,22 was obtained, with the worst evaluations assigned to the recovery of recyclable materials (position 4.7) and minimizing landfill and waste generation (position 4.8). Looking at the examples of actions in Supplementary Table S1, some improvements can be recommended, such as:

- considering the most likely use of chemical/material and if there is the possibility to recycle it,
- avoid using chemical/materials that hamper the recycling processes at EoL,
- selecting processes and materials that minimise the generation of waste,
- selecting materials that are easy to separate and sort.

**Table 6:**
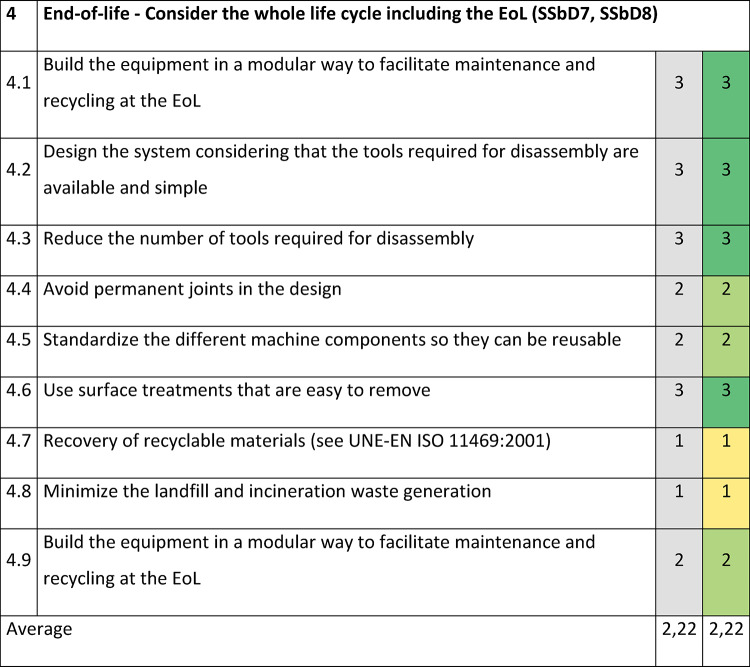
SSbD guiding principles evaluation for the Slurry mixer with mill in Section 4, performed by NETZSCH Feinmahltechnik GmbH.

The scoring is assigned in the colored cells based on the degree of consideration, where 0 = not any consideration, 1 = little, 2 = good, 3 = excellent. Benchmark values are provided in the column shaded in grey.

Finally, aiming to improve the machine manufacturing process, the manufacturer has already conducted a certain number of actions around the design principles previously mentioned. They are presented below, some of them matched with the specific SSbD design principles involved:

- Optimize the process to have less machine components on the system → SSbD1.
- Simple integration of powder handling and technology (integration of dosing system, vacuum, etc.) compared to the reference system → SSbD1.
- Flexible use of the tank volume from 25 to 100% filling level thanks to the conical tank → SSbD1.
- Scalability to bigger production machines from 250 to 20000 L → SSbD1.
- Shortening the mixing time by a factor of 2 to 3 compared to the previous mixer, especially for binder production like PVDF and CMC → SSbD3.
- Higher efficiency and reduced energy costs thanks to the combination of a dispersion mixing tool (primary) and a transport mixing tool (secondary) instead of one mixing tool → SSbD3.
- Reducing the overall complexity to use a modular system → SSbD7.
- Inline rheometric unit.
- Connection to data monitoring system.

## Roll to roll (R2R) and Calendaring unit case study

The evaluation of the design principles on the R2R and calendaring machinery is presented in Tables 7 and 8. Overall, the evaluations are very positive, yielding scores between 2,64 and 2,91 on the scale from 0 to 3.

**Table 7:**
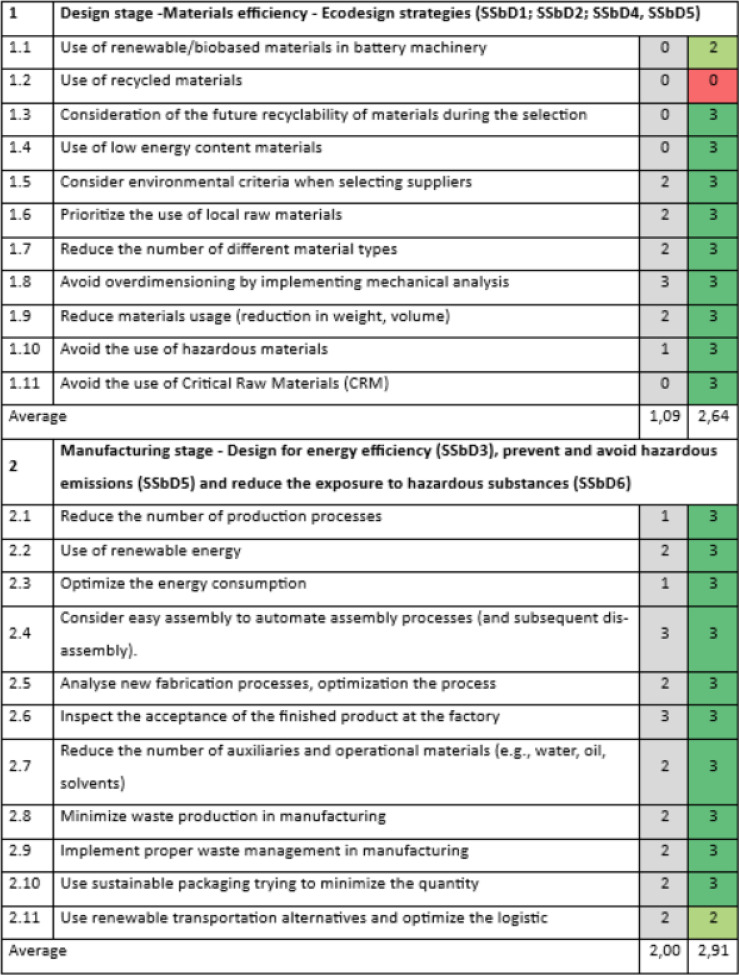
SSbD guiding principles evaluation for R2R and the calendaring unit machines in Sections 1 and 2, performed by FOM TECHNOLOGIES.

The scoring is assigned in the colored cells based on the degree of consideration, where 0 = not any consideration, 1 = little, 2 = good, 3 = excellent. Benchmark values are provided in the column shaded in grey.

**Table 8:**
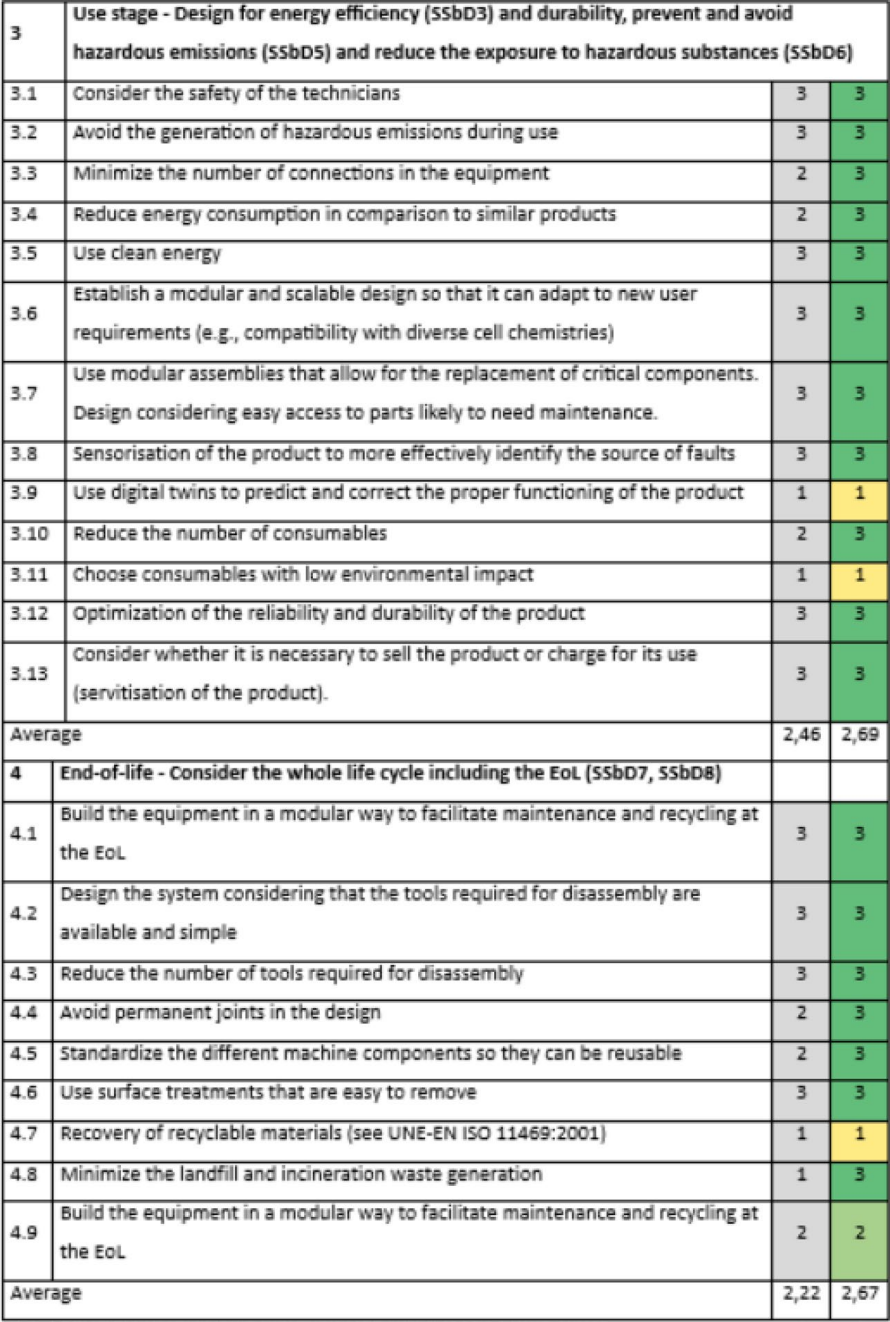
SSbD guiding principles evaluation for R2R and the calendaring unit machines in Sections 3 and 4, performed by FOM TECHNOLOGIES.

The scoring is assigned in the colored cells based on the degree of consideration, where 0 = not any consideration, 1 = little, 2 = good, 3 = excellent. Benchmark values are provided in the column shaded in grey.

The lowest score of 2,64 for Section 1 is related to no consideration of recycled materials during the design stage. Hence, it would be recommended to study the feasibility of using recycled steel in manufacturing, as well as considering new suppliers which can provide products with recycled material in their catalog, without compromising performance. Section 2, which focuses on the manufacturing stage, design for energy efficiency, prevention and avoidance of hazardous emissions and reduction of exposure to hazardous substances, showed the best average score of 2,91. The lowest score in this section is given to the use of renewable transportation alternatives and optimizing logistics (position 2.11), especially related to SSbD3. To improve the design related to this principle, it would be recommended to consider actions aimed at strengthening relations within the supply chain to achieve some synergies and optimization in logistic processes.

Section 3 of the design principles evaluation, related to the use stage and linked to the same principles as Section 2, had an average score of 2,69. The lowest scores correspond to the use of digital twins to predict and correct the proper functioning of the product, together with no choosing of consumables with low environmental impact (positions 3.9 and 3.11). Similar to the mixer and mill case study, it would be preferable to consider the use of digital twins as a long-term objective. On the other hand, it is recommended to identify which are the used consumables that present a higher environmental impact, and try to replace them with others with less impact or, if not possible, look for ways to reduce their use.

Furthermore, several safety and sustainability aspects related to the use stage are already considered in the design of the calendaring unit. These considerations were detailed in a public report^50^ and are presented below, matched with the corresponding SSbD design principles involved:

- Safety precautions and use of PPE: The use of personal protective equipment (PPE) is recommended to minimize exposure to hazardous chemicals → SSbD6.
- Chemical risk assessment: The calendaring process involving certain inks and solvent evaporation has the potential to expose operators to hazardous substances. Operators are advised to consult the safety data sheet for each chemical substance and conduct a comprehensive chemical risk assessment → SSbD6.
- Compliance with standards and normative: Compliance with various safety and design standards ensures that the equipment is safe throughout its life cycle → SSbD6.
- Regular maintenance and cleaning: Detailed instructions for maintenance and cleaning extend the equipment’s lifespan and ensure that materials are handled safely at the end of their life cycle → SSbD1, SSbD7.
- Spare parts management: Classification of spare parts by delivery time helps minimize downtime and optimize resource management → SSbD1.

Section 4 shows the evaluation of end-of-life issues, yielding a score of 2,67. The score reduction is mainly related to low consideration about recovery of recyclable materials (position 4.7). Improvement of this issue would require a better understanding of the possibility of substituting some used materials in terms of recovery after recycling of the equipment.

In summary, the SSbD design principles already integrated in the calendaring unit are focused on material efficiency, risk reduction, and durability.

Concerning the benchmark scores, it was observed that the implementation of the SSbD principles is much more advanced in the R2R and Calendaring unit case study than in the Slurry mixer with mill, especially in Section 1 of the evaluation, addressing the design stage. The smallest difference between the two case studies was found in Section 3, related to the use stage. The evaluations were made at the same time by two machinery manufacturers based in different European countries. Different degrees in the adoption of the SSbD principles were already observed. Almost all sections had average scores between 2 and 3, meaning that in most cases manufacturers have started to implement the SSbD principles with a clearly defined timeline. This illustrates the fact that the adoption of these principles is accelerating in recent years, supported by public investments to reshape Europe’s energy landscape.

### SSbD practice guidelines for battery machinery manufacturing

The present study explored how ecological standards, such as the JRC SSbD framework, can be integrated into the design stage of machinery for battery manufacturing. Through a detailed analysis of design principles and the collection of data on the current design from manufacturers, several key areas have been identified to enhance safety and sustainability in the battery machinery manufacturing process.

As a last compilatory step, the main recommendations for battery manufacturing machinery learned from this work are formulated as follows in Table 9.

**Table 9:** Formulation of the main best practices guidelines recommended for battery cell manufacturing machinery.

1. Increase the use of renewable and recycled materials, considering their future recyclability 2. Reduce the number of different material types to facilitate recycling and reuse 3. Minimize the use of hazardous and critical materials 4. Prioritize the use of local raw materials 5. Reduce the number of production processes and use renewable energy sources 6. Minimize waste generation and manage it properly during manufacturing 7. Implement easy assembly processes and automation to improve energy efficiency, minimize hazardous emissions, and reduce exposure to hazardous substances during the use of the machinery 8. Design machinery in a modular and easy disassembly way to facilitate maintenance, recycling at the end-of-life and adaptation to new user requirements. Reduce the number of tools required for disassembly and standardizing components to improve recyclability 9. Consider the traceability of the product during its use and the identification of its materials and components during disassembly. The future implementation of the DPP in prioritized product categories, such as steel or electronics, can serve as a good tool for addressing this aspect	

In summary, integrating SSbD principles into the design of battery cell manufacturing machinery can significantly improve the safety and sustainability of the process. The recommendations provided in this research should serve as a guide for machinery developers to implement safer and more sustainable design practices.

### Future perspectives, potential trade-offs, and practical barriers to integrate SSbD criteria into early design of battery cell manufacturing machinery

The SSbD framework has a voluntary nature, but it may help to meet regulatory needs, e.g., in the search for alternatives for substances that currently can only be used under time-limited authorisations or exemptions from restrictions. It can also be used as an element in strategically developing a company’s readiness for future legislation that may require the use of safer and more sustainable chemicals, processes and materials to replace substances of concern. After the publication of the SSbD framework in 2022, the EU started to implement SSbD under the Horizon Europe framework programme and intends to continuously refine it. In 2023, the JRC presented the application of the SSBD framework to illustrative case studies^51^, and specific guidance on possible actions to address the challenges found in the implementation of the framework was delivered in 2024^47^. The SSbD framework is expected to be updated in 2025, after considering feedback received from stakeholders and users.

The novelty of this research lies in the fact that it represents a first step for the application of the JRC SSbD framework to battery cell manufacturing machinery. The scoring assigned in the evaluation of the current design principles was based on a degree of consideration by each machinery manufacturer. To fully address the re(design) in the machinery design and innovation stage in the future, we recommend integrating specific indicators into the scoring system, so as the degree of compliance with each design principle can be more accurately quantified. Some examples of indicators related to each SSbD principle have already been defined in the JRC SSbD framework, and further development on this subject can be expected soon.

To implement SSbD guiding principles in practice in real-world manufacturing settings, potential barriers and trade-offs should also be taken into consideration. Some of the challenges identified by the JRC to further improve the SSbD framework^51^ can be assumed as practical barriers for its application to battery machinery design:

- Provide accessible resources to apply the framework (e.g., databases, sector specific guidance, tools), in order to alleviate the high initial cost for its uptake.
- Define the solutions to couple the SSbD assessment with the design and innovation process, avoiding potential compromises between functional performance and safety or sustainability, and difficulties in scalability.
- The standardisation, validation and applicability of models/tools linked with the quality of the data generated needs further assessment. The available metrics for performance measurement are still insufficient.
- Availability of data and information, finding a balance between transparency and reproducibility versus confidentiality, and collaboration across stakeholders for the generation and exchange of data along the supply chains.
- Development of specific skills and training as it requires a wide set of expertise.

To bring SSbD closer to practical applicability in terms of design, Apel et al.^52^ recommended, among others, industry-driven knowledge-sharing hubs for a value chain-specific SSbD ecosystem and the operationalisation of SSbD, especially for small and medium-sized enterprises, and corporate strategy of industry to allow for dialogue between R&D and regulatory and sustainability affairs. Questions and obstacles to fully integrate SSbD principles into practice may also be seen as an opportunity to develop a more realistic and agile framework with clear, simplified methods, and robust support for stakeholders^53^. In addition, current challenges could also be overcome by long-term benefits of integrating SSbD criteria, such as improved market access, and opportunities for process optimization and resource efficiency.

The application of the whole stepwise approach in the JRC SSbD framework, including safety and sustainability assessment and considering different production line configurations and factory designs, will ultimately lead to achieve the best practice proposal for battery cells and manufacturing machinery value chains.

## Supplementary Information

Supplementary Information.

## Data Availability

All data generated or analysed during this study are included in this published article (and its Supplementary Information files).

## Abbreviations

BEV: Battery Electric Vehicle
CRM: Critical Raw Materials
EC: European Commission
EC-CSS: European Chemical Strategy for Sustainability
EoL: End-of-life
ESS: Energy storage systems
JRC: Joint Research Center
LCA: Life Cycle Assessment
LCT: Life Cycle Thinking
LFP: Lithium Iron Phosphate
LIB: Lithium-ion batteries
NMC: Lithium Nickel Manganese Cobalt oxide
R2R: Roll to roll
S-LCA: Social Life Cycle Assessment
SSbD: Safe and Sustainable by Design
SVHC: Substances of very high concern
