# Supplementary material for: Integration of safety and sustainability criteria into early design stages of battery cell manufacturing machinery
Source: Sci Rep. 2025 Dec 19;16:825. doi: 10.1038/s41598-025-30510-7 (PMC12780080; doi:10.1038/s41598-025-30510-7)
Supplement: Supplementary file 1 — Supplementary Information. [file 41598_2025_30510_MOESM1_ESM.docx]

**SUPPLEMENTARY INFORMATION**

**Integration of safety and sustainability criteria into early design stages of battery cell manufacturing machinery**

Cristina Cerrillo^1^, Gemma Mendoza^1^, Guillermo Ormazabal^1^, Vladimir Popok^2^, Heiko Oetting^3^, Benedikt Konersmann^4, 5^, Alessandro Tedeschi Gallo^6^, Maeva Lavigne Philippot^7^

^1^ Tekniker, Basque Research and Technology Alliance (BRTA), C/ Iñaki Goenaga, 5, 20600, Eibar, Spain

^2^ FOM TECHNOLOGIES, Kastrup, Denmark

^3^ NETZSCH Feinmahltechnik, Selb, Germany

^4^ RWTH AACHEN UNIVERSITY, Aging and Lifetime Prediction of Batteries, Institute for Power Electronics and Electrical Drives (ISEA), Campus-Boulevard 89, 52074 Aachen, Germany

^5^ Helmholtz-Institute Münster (HI MS), Institute of Energy Materials and Devices (IMD-4), Forschungszentrum Jülich GmbH, Campus-Boulevard 89, 52074 Aachen, Germany

^6^ Deep Blue, Rome, Italy

^7^ Electromobility Research Center (MOBI), Department of Electric Engineering and Energy Technology (ETEC), Vrije Universiteit Brussel, Pleinlaan 2, 1050, Brussels, Belgium

Corresponding Author: Cristina Cerrillo. Email address: [cristina.cerrillo@tekniker.es](mailto:cristina.cerrillo@tekniker.es)

Search terms used to on the Web of Science to conduct peer-reviewed papers search:

- "safe and sustainable by design" or "ssbd" (All fields) AND "batter*" and "lithium" (All fields) AND "machine*" and "manufactur*" (All fields) AND 2020-01-01 to 2025-02-01 (Publication date).
- "safe and sustainable by design" or "ssbd" (All fields) AND "batter*" (All fields) AND "machine*" or "equipment" or "manufactur*" (All fields) AND 2020-01-01 to 2025-02-01 (Publication date).
- "safe and sustainable by design" or "ssbd" (All fields) AND "batter*" or "machine*" or "equipment" (All fields) AND 2020-01-01 to 2025-02-01 (Publication date).
- "sustainab*" or "ssbd" (All fields) AND "batter*" and "machine*" and "equipment" and "manufactur*" (All fields) AND 2020-01-01 to 2025-02-01 (Publication date).
- "sustainab*" or "ssbd" (All fields) AND "batter*" and "lithium" and "machine*" and "manufactur*" (All fields) AND 2020-01-01 to 2025-02-01 (Publication date).

Table S1. List of SSbD design principles defined by the JRC framework and associated definition, and examples of actions that can be used in the design phase [5].

| SSbD principle | Definition | Examples of actions |
| --- | --- | --- |
| SSbD1 Material efficiency | Pursuing the incorporation of all the chemicals/materials used in a process into the final product or full recovery inside the process, thereby reducing the use of raw materials and the generation of waste. | - Maximise yield during reaction to reduce chemical/material consumption  - Improve recovery of unreacted chemicals/materials  - Optimise solvent for purpose (amount, typology and recovery rate)  - Select materials and processes that minimise the generation of waste  - Minimise the number of chemicals used the production process  - Minimize waste generation  - Identify occurrence of use of Critical Raw Material, towards minimizing or substituting them |
| SSbD2 Minimise the use of hazardous chemicals /materials | Preserve functionality of products while reducing or completely avoid using hazardous chemicals/materials where possible. | - Reduce and/or eliminate hazardous chemicals/materials in manufacturing processes  - Verify possibility of using hazardous chemicals/materials in close loops when they cannot be reduced or eliminated  - Eliminate hazardous chemical/materials in final products |
| SSbD3 Design for energy efficiency | Minimise the overall energy used to produce a chemical/material in the manufacturing process and/or along the supply chain. | Select and/or develop (production) processes considering:  - Alternative and lower energy intensive production/separation techniques  - Optimize energy efficiency of solvent recovery  - Maximise energy re-use (e.g. heat networks integration and cogeneration)  - Fewer production steps (e.g. applying lean thinking)  - Use of catalysts, including enzymes  - Reduce inefficiencies and exploit available residual energy in the process or select lower temperature reaction pathways |
| SSbD4 Use renewable sources | Target resource conservation, either via resource closed loops or using renewable material/ secondary material and energy sources. | Verify the possibility of selecting feedstocks that:  - are renewable or secondary materials  - do not create land competition  and/or processes that:  - use energy resources which are renewable and with low carbon emissions |
| SSbD5 Prevent and avoid hazardous emissions | Apply technologies to minimise and/or to avoid hazardous emissions or pollutants in the environment. | Select materials and/or processes that:  -minimise the generation of hazardous waste  -minimise generation of emissions (e.g. Volatile Organic Compounds, acidifying and eutrophying pollutants, heavy metals, etc.) |
| SSbD6 Reduce exposure to hazardous substances | Eliminate exposure to chemical hazards from processes as much as possible. Substances which require a high degree of risk management should not be used and the best technology should be used to avoid exposure along all the life cycle stages. | - Eliminate or minimise risk through reduction of the use of hazardous substances  - Analyse and avoid as much as possible the use of substances identified as SVHC  - Consider value chain-specific regulations  - Reduction and/or elimination of hazardous substances in manufacturing processes |
| SSbD7 Design for end-of-life | Design chemicals/materials in a way that, once they have fulfilled their function, they break down into products that do not pose any risk to the environment/humans.  Design for preventing the hindrance of reuse, waste collection, sorting and recycling/upcycling. | - Avoid using chemical/materials that hamper the recycling processes at EoL  - Select processes (and material) that minimise the generation of waste.  - Select materials that are (where appropriate):   - more durable (extended life and less maintenance) - easy to separate and sort - valuable after their use (commercial after life) - truly biodegradable for uses which unavoidably lead to dispersion into the environment or wastewater |
| SSbD8 Consider the whole life cycle | Apply the other design principles thinking through the entire life cycle, from supply-chain of raw materials to the EoL in the final product. | Consider for example:  - Using reusable packaging for the chemical/material under assessment and for chemicals/materials in its supply-chain  - Consider the most likely use of chemical/material and if there is the possibility to recycle it  - Energy-efficient logistics (i.e. reduction of transported quantities, change in mean of transport)  - Reducing transport distances in the supply-chain  - Applying responsible sourcing principles |

Table S2. List of Critical Raw Materials and Strategic materials for the EU in 2023 [46].

| **2023 Critical Raw Materials (*Strategic Raw Materials in italics)*** | | | |
| --- | --- | --- | --- |
| aluminium/bauxite  antimony  arsenic  baryte  beryllium  *bismuth*  *boron*/borate  *cobalt* | coking coal  feldspar  fluorspar  *gallium*  *germanium*  hafnium  helium  *HREE* | *lithium*  LREE  *magnesium*  *manganese*  *natural graphite*  niobium  *PGM*  phosphate rock  *copper** | phosphorus  scandium  *silicon metal*  strontium  tantalum  *titanium metal*  *tungsten*  vanadium  *nickel** |

*Copper and nickel do not meet the CRM thresholds but are included as Strategic Raw Materials.

Table S3. List of aspects (hazard properties) relevant for Step 1 in the EC JRC SSbD framework [5] (references included in this source have been removed).

| Group definition | Human health hazards | Environmental hazards | Physical hazards |
| --- | --- | --- | --- |
| Includes the most harmful substances (according to CSS), including the substances of very high concern (SVHC) according to REACH Art. 57(a-f).  These hazard properties form Criterion H1. | - Carcinogenicity Cat. 1A and 1B. - Germ cell mutagenicity Cat. 1A and 1B. - Reproductive / developmental toxicity Cat. 1A and 1B - Endocrine disruption Cat. 1 (human health) - Respiratory sensitisation Cat. 1 - Specific target organ toxicity - repeated exposure (STOT-RE) Cat. 1, including immunotoxicity and neurotoxicity | - Persistent, bioaccumulative and toxic / very persistent and very bioaccumulative (PBT/vPvB) - Persistent, mobile and toxic / very persistent and mobile (PMT/vPvM) - Endocrine disruption Cat. 1 (environment) | - NA |
| Includes substances of concern, as described in CSS, defined in the Article 2(28) of SPI proposal and that are not already included in Criterion H1.  These hazard properties form Criterion H2. | - Skin sensitisation Cat 1 - Carcinogenicity Cat. 2 - Germ cell mutagenicity Cat. 2 - Reproductive / developmental toxicity Cat. 2 - Specific target organ toxicity - repeated exposure (STOT-RE) Cat. 2 - Specific target organ toxicity - single exposure (STOT-SE) Cat. 1 and Cat. 2 - Endocrine disruption (human health) Cat. 2 | - Hazardous for the ozone layer - Chronic environmental toxicity (chronic aquatic toxicity) - Endocrine disruption Cat. 2 (environment) | - NA |
| Includes the other hazard classes not part already in Criteria H1 and H2.  These hazard properties form Criterion H3. | - Acute toxicity - Skin corrosion - Skin irritation - Serious eye damage/eye irritation - Aspiration hazard Cat. 1 - Specific target organ toxicity - single exposure (STOT-SE) Cat. 3 | - Acute environmental toxicity (acute aquatic toxicity) | - Explosives - Flammable gases, liquids and solids - Aerosols - Oxidising gases, liquids, solids - Gases under pressure - Self-reactive - Pyrophoric liquids, solid - Self-heating - In contact with water emits flammable gas - Organic peroxides - Corrosivity - Desensitised explosives |

Table S4. Description of criteria related to Step 1 [5].

| Criteria | Description | Observations  (in alignment with EC-CSS) |
| --- | --- | --- |
| Criterion H1 | The criterion refers to the most harmful substances, according to EC-CSS, including the SVHC according to REACH Art. 57(a-f) and additional hazard properties, as defined in Supplementary Table S3.  This is a cut-off criterion, establishing a minimum set of hazard requirements that need to be fulfilled by a chemical or material in order to be considered eventually SSbD after the other assessments are performed.  Therefore, the assessment of the other aspects can be performed in order to understand the overall SSbD performance (e.g., safety during the use assessed in Step 3, other environmental sustainability aspects assessed in Step 4) if this helps the innovation process. | The chemicals and materials which do not pass this criterion should be:  - Prioritised for substitution  - Re-designed in order to reduce their adverse effects  - Only allowed in uses proven essential for society (e.g. if their use is necessary for health, safety or is critical for the functioning of society and if there are no alternatives that are acceptable from the standpoint of environment and health)  - Safely used and emissions/exposure be controlled along the whole life cycle while activities are undertaken to develop alternatives as soon as possible and their use is phased out as soon as less hazardous alternatives are available  - Tracked through their life cycle |
| Criterion H2 | The criterion refers to the hazard class categories and hazardous substances which are part of the substances of concern described in EC-CSS and not included already in criterion H1, as defined in Supplementary Table S3.  For the chemicals or materials with hazard properties a safety level or score will be assigned, while the SSbD assessment will continue with the evaluation of the other safety and sustainability aspects, in order to assess their overall SSbD performance. | The chemicals and materials that do not pass this criterion should be:  - Substituted as far as possible  - Re-designed in order to reduce their adverse effects  - Safely used and emissions/exposure be controlled along the whole life cycle, until less hazardous alternatives are available  - Tracked through their life cycle |
| Criterion H3 | The criterion refers to the group of other hazard classes, including here all hazard properties not covered by criteria H1 and H2, as defined in Supplementary Table S3.  Following a similar approach described above, a safety level or score will be assigned to the chemicals or materials under this category in order to be integrated in the overall SSbD assessment. | The chemicals and materials that do not pass this criterion should be:  - Flagged for review and eventually reduce toxic effects  - Ensure their safety along the life cycle until less hazardous alternatives are available |
